# The inflammation link between periodontal disease and coronary atherosclerosis in patients with acute coronary syndromes: case–control study

**DOI:** 10.1186/s12903-020-01356-4

**Published:** 2021-01-06

**Authors:** Agnieszka Wojtkowska, Tomasz Zapolski, Joanna Wysokińska-Miszczuk, Andrzej P. Wysokiński

**Affiliations:** 1 Department of Cardiology, Medical University of Lublin, ul. Jaczewskiego 8, 20-954 Lublin, Poland; 2 Department of Periodontology, Medical University of Lublin, Lublin, Poland

**Keywords:** Myocardial infarction, Coronary atherosclerosis, Periodontal disease, Inflammation

## Abstract

**Background:**

Coronary atherosclerosis and periodontal disease, due to their prevalence, are a serious epidemiological problem. Pathophysiological evidence points to their possible common inflammatory etiopathological background. The aim of the study was to analyze the relationship between the presence and severity of periodontitis, systemic inflammation and selected parameters of myocardial injury and heart function in patients with acute myocardial infarction.

**Methods:**

The study group consisted of 71 patients 54.22 (7.05)-year-old hospitalized due to acute myocardial infarction. The patients underwent a coronary angiographic examination and echocardiography. The following laboratory parameters were determined: blood morphology, high sensitivity C-reactive protein (hsCRP), erythrocyte sedimentation rate (ESR), fibrinogen, troponin I, creatine kinase myocardial band (CK-MB), brain natriuretic peptide (BNP), lipidogram, glucose, creatinine, glomerular filtration rate (GFR), thyroid stymulating hormone (TSH), glycated hemoglobin (HbA1c). Dental assessment of the patients was performed and the following indicators were included: the number of teeth preserved, approximal plaque index (API), bleeding on probing (BoP), pocket depth (PD), the number of bleeding periodontal pockets ≥ 4 mm in depth (NoPD ≥ 4 mm), the percentage of bleeding periodontal pockets ≥ 4 mm in depth (%PD ≥ 4 mm), clinical attachment loss (CAL). The control consisted of 40 patients 52 (± 8.43)-year-old without a history of coronary heart disease. These patients were subjected to a periodontal examination using the above parameters and classification methods. The following statistical tests were implemented: Shapiro–Wilk test, Levene's test, Mann Whitney's U analysis, Univariate Analysis of Variance (ANOVA); the post-hoc analysis was performed with the use of Tukey's honest significant difference test (HSD), Kruskal–Wallis's non-parametric test, Spearman's rank correlation, logistic regression analysis, linear regression analysis and ROC analysis.

**Results:**

The BoP (bleeding on probing) significantly correlated with fibrynogen (R-0.36; *p*-0.006). All indices regarding the pocket depth correlated significantly with the number of leukocytes: PD (R-0.27; *p*-0.02), NoPD ≥ 4 mm (R-0.28, *p*-0.02), %PD ≥ 4 mm (R-0.27; *p*-0.02). PD (R-0.28; *p*-0.01) and NoPD ≥ 4 mm (R-0.24; *p*-0.04) were also associated significantly with the level of hsCRP. The BoP is correlated closely with the levels of BNP (R-0.29, *p*-0.02). The multifactorial analysis showed that significant predictors of myocardial infarction are API and BoP. The analysis showed that API and BoP are important predictors of troponin levels. Linear regression analysis showed that only CAL is a significant predictor of BNP.

**Conclusions:**

Patients with acute myocardial infarction have worse periodontal status compared to people without coronary heart disease. Greater severity of periodontitis, plaque accumulation and bleeding on probing are associated with acute myocardial infarction. Periodontitis is a risk factor for myocardial infarction and also affects the degree of post-infarction left ventricular damage, which means that there is an inflammatory link between these two diseases.

## Background

Due to their prevalence, cardiovascular diseases are considered as social diseases; they are also among the most common causes of death worldwide [1]. Periodontal diseases, in turn, belong to the most common illnesses of the oral cavity and, along dental caries, constitute the main cause of teeth loss [2, 3]. Periodontal infections are also linked to the risk pattern of several systemic diseases such as cardiovascular diseases or rheumatoid arthritis [4]. In a study investigating the association between oral hygiene and cardiovascular disease, data from the National Health Insurance System—National health Screening Cohort (NHISHEALS) including 247.696 individuals with no history of cardiovascular disease from 2002 to 2003, the presence of periodontal diseases was related to greater tooth loss, which, in turn, was associated with increased risk of future major cardiovascular events, including death, acute myocardial infarction, heart failure and stroke [5]. The common factors contributing to the incidence of both cardiovascular disease and chronic periodontal disease include ageing, smoking, alcohol abuse, race/ethnicity, education and socioeconomic status, male sex, diabetes mellitus, and overweight or obesity [5, 6]. Scientific evidence also indicates the presence of genetic factors in periodontitis and cardiovascular diseases [7].

Periodontitis is preceded by gingival inflammation (gingivitis), which is reversible if a proper therapy is employed. As a result of inflammation of the periodontium, tissues surrounding the tooth are infiltrated by neutrophils, macrophages and, subsequently, by activated lymphocytes, releasing, e.g., interleukin-1, prostaglandin E_2_, and tumour necrosis factor-α [8]. A considerably large area of the periodontal tissue, as well as its dense vascularisation, enables bacteria, bacterial metabolic products, and inflammatory mediators to enter the bloodstream and thus promotes endothelial dysfunction [9].

It has been known for a while that periodontitis alone is an independent risk factor contributing to the development of atherosclerotic vascular disease and the underlying mechanism is systemic inflammation [10]. Atherosclerosis is a chronic inflammatory process affecting the intima of mainly large and medium caliber arteries, leading to formation of lipid build-ups created as a result of accumulation of inflammatory cells and the formation of a fibro-lipid structure, referred to as atherosclerotic plaque. Theories regarding the pathogenesis of atherosclerosis have changed over time. Currently, the role of autoimmune and inflammatory conditions in the onset and progression of atherosclerosis and in developing its complications is underlined. Endothelial dysfunction resulting from immune and inflammatory reactions within the vessel wall is the earliest and most important process in the development of atherosclerosis [11, 12].

Coronary artery disease, connected to atherosclerotic inflammation, and acute coronary syndromes are conditions resulting in the increase of both C Reactive Protein (CRP) levels and leukocytosis [13]. Leukocytes take part in atherosclerosis through their participation in the inflammatory process; they cause endothelial dysfunction, microcirculation disorders, have proteolytic, oxidative, and procoagulant effect.

The mechanisms with which active inflammatory lesions in the periodontal tissue influence the blood vessel wall are still in the scope of numerous researches. The immune and inflammatory background of those dependencies is emphasized. Among a few complementary hypotheses, there are two most important ones; the first one stipulates that bacteria and their toxins have a direct impact on the vessel wall during bacteremia; the second one assumes that cytokines and inflammatory mediators released during chronic periodontal inflammation may potentially affect the vessel wall [14, 15]. Despite many data supports the relationship between periodontitis and atherosclerosis, which are two common civilization pathologies, still too little attention is paid to the search for their joint etiopathogenesis to limit their adverse social effects.

## Aim of study

The main goal was to analyze the relationship between the presence and severity of periodontitis and selected parameters of myocardial injury and heart function in patients with acute myocardial infarction. In addition, the impact of periodontitis on selected laboratory and morphological parameters of inflammation was analyzed in patients with acute myocardial infarction and in the group of people without confirmed ischemic heart disease.

## Methods

### Study sample

**Fig. 1:**
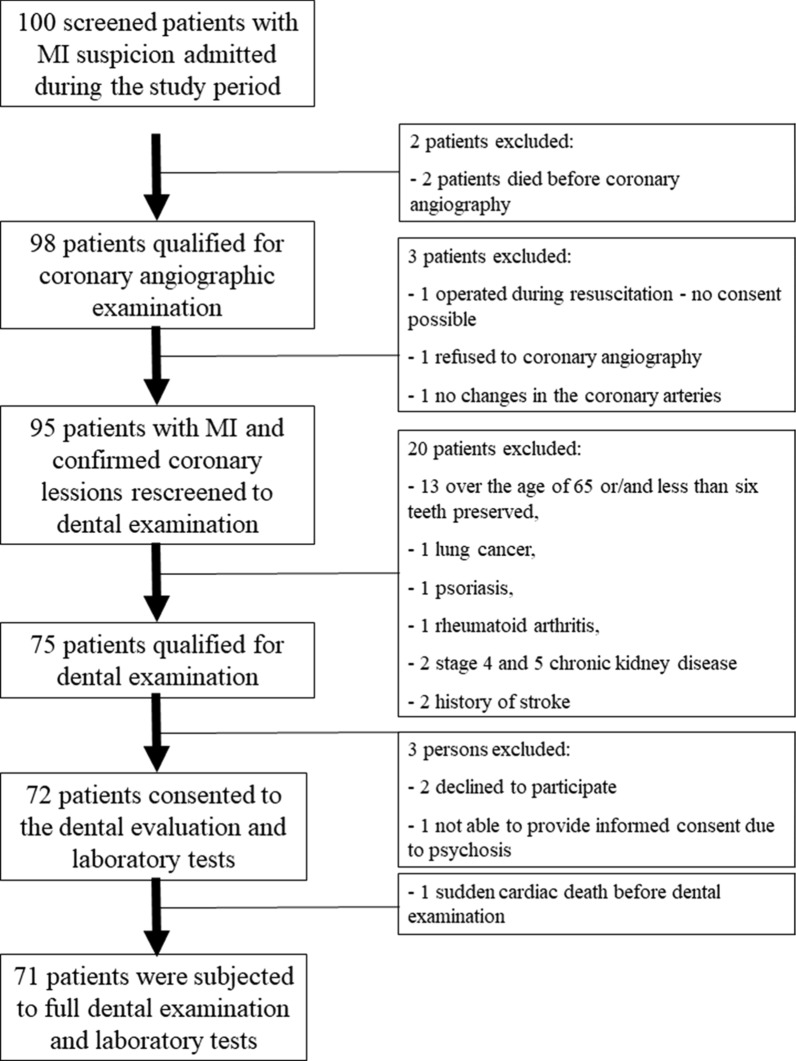
Study group participants diagram of flux

Study group was composed by 71 patients, both gender, < 65 years, hospitalized due to acute myocardial infarction. Exclusion criteria were: < 6 remaining teeth, diagnosis of neoplasms, autoimmune diseases, rheumatological diseases, chronic kidney diseases stages 4 and 5 and history of cerebrovascular accident (Fig. 1).

**Fig. 2:**
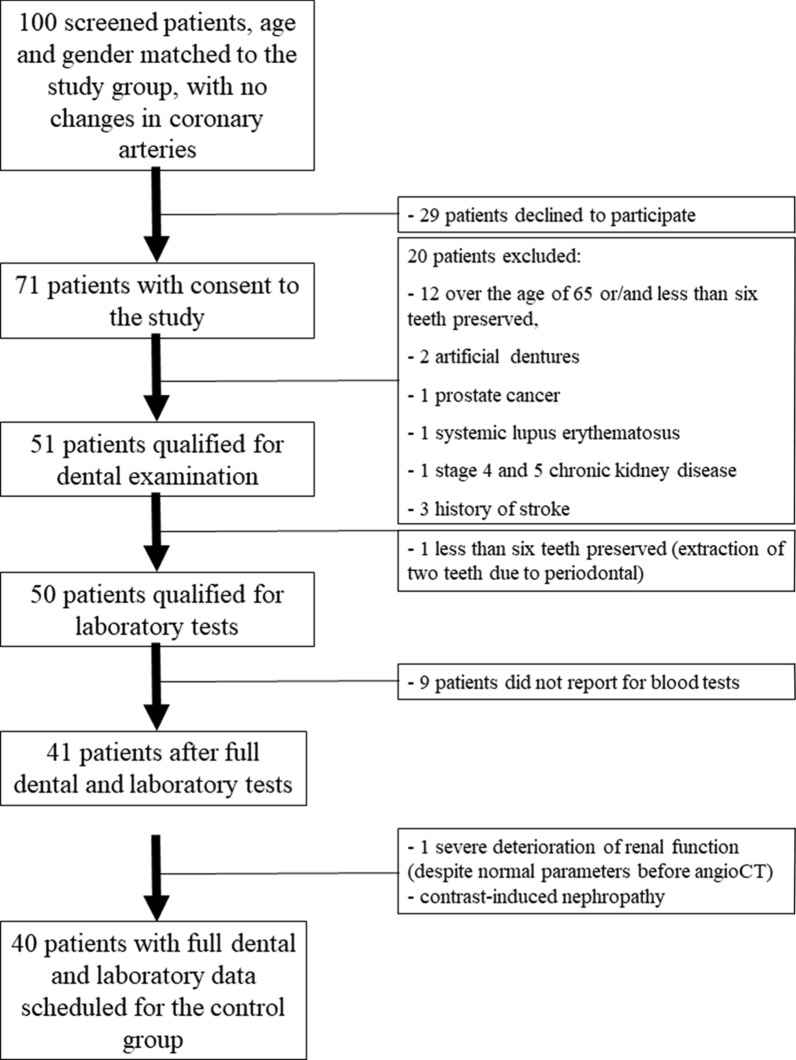
Control group participants diagram of flux

Control group was composed by 40 patients, both gender, < 65 years, with no history of coronary heart disease, as determined by a 64-slice multidetector computed ambulatory examinations (Fig. 2).

The patients in the study group were recruited from the Cardiology Department of Medical University of Lublin. The patients in the control group were recruited from among patients who attended the Cardiology Clinic at the Department of Cardiology for the diagnosis of coronary heart disease. The recruitment period covered: September 2018–July 2019.

All patients were subjected to periodontal examination and answered a health questionnaire investigating risk factors for ischemic heart disease, such as: nicotinism, hypertension, diabetes, lipid disorders and smoking. Patients were measured for height and weight and their body mass index (BMI) was determined; BMI in the range 25–29.9 kg/m^2^ was defined as overweight and ≥ 30 kg/m^2^ as obesity.

The study was approved by the Bioethical Committee of the Medical University of Lublin (decision number: KE-0254/58/2016). All patients included in the study were fully informed and gave written consent to the participation. The study protocol conforms to the ethical guidelines of the 1975 Declaration of Helsinki.

### Cardiology assessment

According to the present guidelines the acute myocardial infarction (MI) were recognized in patients with chest discomfort or other ischaemic symptoms, who develop new ST segment elevations in two contiguous leads or new bundle branch blocks with ischaemic repolarization patterns as an ST-elevation MI (STEMI) or without ST-segment elevation (NSTEMI) and the presence of acute myocardial injury detected by abnormal cardiac biomarkers in the setting of evidence of acute myocardial ischaemia [16].

In the study group, the patients underwent a standard coronary angiographic examination on an Innova 4100 angiograph (GE Healthcare, Waukesha, WI). After the evaluation of coronary arteries, patients underwent revascularization in accordance with current standards [17, 18]. Echocardiographic tests were performed with a Philips iE33 (Koninklijke Philips Electronics NV) in accordance with the principles of the American Echocardiographic Society, assessing the following echocardiographic parameters: left ventricle enddiastolic diameter, left ventricle endsystolic diameter, left ventricle ejection fraction (by the Simpson method), left atrium maximal diameter, right ventricle enddiastolic diameter [19].

### Laboratory data

The following laboratory parameters were determined: blood morphology: hemoglobin, hematocrit, red cells count, leukocytes count, platelets count; inflammation parameters: high sensitivity C-reactive protein (hsCRP), erythrocyte sedimentation rate (ESR), neutrofiles, lymphocytes, neutrofiles to lymphocytes ratio (N/L ratio), fibrynogen; parameters of myocardial injury: troponin I (TnI), creatine kinase myocardial bound (CK-MB); parameters of heart failure: brain—type natriuretic peptide (BNP); lipidogram: total cholesterol, low-density lipoprotein cholesterol (LDL-cholesterol), high-density lipoprotein cholesterol (HDL-cholesterol), triglycerides (TG); glucose, creatinine, glomerular filtration rate (GFR), thyroid-stimulating hormone (TSH), glycated hemoglobin (HbA_1C_).

Blood counts were determined by flow cytometry using an ADVIA 2120i apparatus (Siemens); fibrinogen level by measuring the clotting time using the ACL TOT 500 apparatus of the Instrumentation Laboratory. The lipid profile was determined by the enzymatic method, HDL-cholesterol level by direct elimination with catalase, TG level by spectrophotometric enzymatic reaction, CRP level by the immunoturbidimetric method using latex, HbA_1C_ by the immunoturbidimetry method, glucose level by the hexokinase method, creatinine by the Jaffe method with alkaline picrate. These tests were performed with the ADVIA 1800 apparatus from Siemens. BNP and TSH levels were determined by direct chemiluminescence using the ADVIA Centaur XP apparatus from Siemens. TnI in whole blood was determined by electrochemiluminescence using a Roche Elecsys 2010 analyzer (Roche Diagnostics GmbH, Mannheim, Germany). The minimum detection level was 0.01 µg/l.

### Dental examination

Dental assessment of the patients was performed on the bedside on the next day after the admission to the Department of Cardiology. In order to ensure the uniformity and repeatability of the assessment, an oral examination was performed by one dentist. Dental examination was performed with the use of artificial lighting, with a dental mirror and a Hu-Friedy PCPUNC 15 perio probe. Third molars were excluded from the examination. Patients enrolled in the study had to have at least 6 teeth. The following indicators were included in periodontal assessment:

- the number of teeth preserved,
- approximal plaque index (API) by Lange [20],
- bleeding on probing (BoP) by Ainamo and Bay [21],
- pocket probing depth (PD), measured from gingival margin to the bottom of the sulcus or pocket,
- the number of bleeding periodontal pockets ≥ 4 mm in depth (NoPD ≥ 4 mm),
- the percentage of bleeding periodontal pockets ≥ 4 mm in depth (%PD ≥ 4 mm),
- clinical attachment loss (CAL), determined as the distance from cementum-enamel junction and the bottom of the sulcus or pocket.

The parameters were assessed at four points: mesiobuccal, buccal, distobuccal, and lingual.

Periodontitis has been categorized according to the American Academy of Periodontology (AAP) classification updated in 2017. It was staged based on severity and complexity of management for [22]:

- Stage I—incipient periodontitis
- Stage II—moderate periodontitis
- Stage III—periodontitis with potential for additional tooth loss
- Stage IV—advanced periodontitis with extensive tooth loss and potential loss of dentition.

### Statistical analysis

The obtained data were analysed with the use of Microsoft Office 2007 and StatSoft Statistica v. 10.0. In the statistical description of the obtained results, the average, median, minimum, and maximum values were used. Normal distribution of data was tested with the Shapiro–Wilk test, while homogeneity of variance was estimated with the Levene's test. To compare the control and study groups, Mann Whitney's U analysis was performed. The concentrations of the studied parameters were tested with the Univariate Analysis of Variance (ANOVA); the post-hoc analysis was performed with the use of Tukey's honest significant difference test (HSD). In the case of a lack of homogeneity of variance, Kruskal–Wallis's non-parametric test was used. The correlation between the parameters was described with Spearman's rank correlation coefficient.

Logistic regression analysis was performed to determine the prediction model of myocardial infarction. To determine the prediction of troponin, BNP and left ventricular ejection fraction levels, linear regression analysis was performed. The inflation variance coefficient was assessed for all parameters included in the linear regression analysis. Thus, the value of this coefficient allowed to determine whether a given predictor was correlated with other predictors in the model. The lack of collinearity of predictors in the model was assumed in cases when the coefficient reached the value of ≤ 4, and the parameter was included in the analysis. However, when the value of the coefficient was ≥ 5, it indicated the possibility of collinearity between the predictors, consequently the parameter was excluded from the analysis. The ROC analysis was applied to assess the quality of the classification of the occurrence or the lack of occurrence of a myocardial infarction.

Moreover, the differences found were considered statistically significant at the *p* level < 0.05.

## Results

### Basic characteristics

The study group consisted of 71 people (56 [79%] men and 15 [21%] women) hospitalized due to myocardial infarction. The average age in the study group was 54 (from 36 to 65 years of age). The clinical characteristics of the study group are shown in Table 1. On the basis of the markers of myocardial necrosis in serum samples (TnI, CK, CK-MB, and myoglobin) and electrocardiography, according to the accepted standards, non-ST-elevation myocardial infarction was recognized in 30% of the patients, while ST elevation myocardial infarction in 70%. The patients underwent coronary angiography. In coronary angiography, 39% of patients had significant coronary stenosis in one coronary artery, while 61% of patients had a multivessel disease. Hemodynamically significant atherosclerotic plaques were described in 3% of cases in the left main coronary artery, in 75% of patients in the left anterior descending artery, in 51% in the circumflex artery and 48% of patients in the right coronary artery (61% of patients had stenosis in more than one vessel). The majority of the patients (97%) were subjected to an invasive treatment, i.e. percutaneous coronary intervention (n-66) or coronary artery bypass grafting (n-3). The remaining patients were provided with conservative treatment (n-2).

**Table 1:** Comparative characteristics of selected parameters in the study group and control group

Parameter	Study group (N = 67)		Control group (N = 40)	*p*
*Anthropometry data*				
Age [years]	54.22 (± 7.05)		52 (± 8.43)	NS
Weight [kg]	84.17 (± 15.43)		72.47 (± 16.84)	NS
Height [cm]	171.90 (± 8.22)		169.71(± 9.59)	NS
BMI [kg/m^2^]	28.15 (± 4.21)		25.04 (± 5.21)	< 0.05
*Medical history*				
Hypertension [N(%)]	45 (68%)		2 (5%)	< 0.05
Diabetes [N(%)]	16 (24%)		2 (5%)	< 0.05
Dyslipidemia [N(%)]	36 (53%)		5 (12.5%)	< 0.05
Smoking [N(%)]	48 (72%)		26 (65%)	NS
Previous myocardial infarction [N(%)]	11 (17%)		0 (0%)	< 0.05
Peripheral artery disease [N(%)]	2 (3%)		0 (0%)	< 0.05
Family history of cardiovascular disease [N(%)]	12 (18%)		5 (12.5%)	< 0.05
*Pressure value*				
Systolic blood pressure [mmHg]	139.42 (± 28.35)		138.75 (± 31.43)	NS
Diastolic blood pressure [mmHg]	85.23 (± 15.6)		84.22(± 16.43)	NS
*Medication*				
Aspirin [N(%)]		64 (96%)	5 (12.5%)	< 0.05
P_2_Y_12_ inhibitors [N(%)]		64 (96%)	0 (0%)	< 0.05
Beta-blockers [N(%)]		60 (90%)	10 (25%)	< 0.05
ACE-inhibitors [N(%)]		49 (73%)	4 (10%)	< 0.05
Sartans [N(%)]		13 (20%)	0 (0%)	< 0.05
Statins [N(%)]		67 (100%)	0 (0%)	< 0.05
Fibrats [N(%)]		0 (0%)	0 (0%)	< 0.05
Diuretics [N(%)]		5 (7%)	0 (0%)	< 0.05
Nonsteroidal anti-inflammatory drugs [N(%)]		0 (0%)	0 (0%)	NS
*Laboratory data*				
Inflammation parameters				
hsCRP [mg/l]	8.34 (± 18.72)		1.23 (± 3.55)	< 0.05
ESR [mm/h]	28.69 (± 17.62)		5.01 (± 3.42)	< 0.05
Leukocytes [10^9^/l]	10.82 (± 3.87)		6.48 (± 2.57)	< 0.05
Neutrophiles [%]	57.3 (± 5.7)		53.2 (± 3.8)	NS
Lymphocytes [%]	10.5 (± 4.0)		12.4 (± 4.5)	NS
N/L ratio [n]	4.12 (± 2.85)		4.29 (± 2.01)	< 0.05
Fibrinogen [g/l]	4.62 (± 1.09)		4.11 (± 1.11)	< 0.05
Parameters of myocardial injury				
TnI [ng/ml]	31.24 (± 20.40)		0.19 (± 0.09)	< 0.05
CK-MB [U/l]	126.53 (± 111.06)		9.34 (± 3.22)	< 0.05
Parameters of heart failure				
BNP [pg/ml]	145.43 (± 181.31)		64.96 (± 22.62)	< 0.05
Lipidogram				
Total cholesterol [mg/dl]	208.96 (± 50.72)		189.36 (± 23.94)	< 0.05
LDL-cholesterol [mg/dl]	131.01 (± 45.56)		111.83 (± 34.65)	< 0.05
HDL-cholesterol [mg/dl]	46.8 (± 13.34)		47.89 (± 11.83)	NS
TG [mg/dl]	171.36 (± 107.88)		145.52 (± 45.31)	< 0.05
Glucose [mg/dl]	145.01 (± 65.66)		98.11 (± 22.34)	< 0.05
Blood morphology				
Hemoglobin [g/dl]	13.2 (± 1.14)		13.6 (± 1.23)	NS
Hematocrit [%]	43.3 (± 4.9)		44.1 (± 4.6)	NS
Red cells count [10^12^/l]	4.11 (± 0.31)		4.32 (± 0.29)	NS
Platelets count [10^9^/l]	176.6 (± 70.1)		173.5 (± 54.50)	NS
Other				
Creatinine [mg/dl]	0.85 (± 0.21)		0.82 (± 19.2)	NS
GFR [ml/min/1.73 m^2^]	62.4 (± 14.7)		78.9 (± 12.4)	< 0.05
TSH [mU/l]	1.78 (± 3.57)		1.65 (± 2.91)	NS
HbA_1_C [%]	6.03 (± 1.14)		5.51 (± 0.52)	< 0.05
*Coronarography results*				
Right coronary artery [N(%)]	32 (48%)		NA	
Left main coronary artery [N(%)]	2 (3%)		NA	
Left anterior descending artery [N(%)]	50 (75%)		NA	
Circumflex artery [N(%)]	34 (51%)		NA	
*Echocardiography data*				
Left ventricle end diastolic diameter [mm]	52.1 (± 4.5)		51.1 (± 4.9)	NS
Left ventricle end systolic diameter [mm]	10.8 (± 1.12)		10.42 (± 1.09)	NS
Left ventricle ejection fraction [%]	54.58 (± 6.95)		65.09 (± 6.32)	< 0.001
Left atrium maximal diameter [mm]	39.5 (± 4.1)		37.6 (± 3.9)	NS
Right ventricle end diastolic diameter [mm]	2.84 (± 1.09)		2.67 (± 1.21)	NS
*Parameters from dental examination*				
Number of preserved teeth [n]	17.28 (± 6.42)		23.53 (± 5.46)	< 0.001
API [%]	77.68 (± 26.24)		39.44 (± 27.93)	< 0.001
BoP [%]	42.99 (± 23.88)		44.42 (± 26.36)	NS
PD [mm]	2.64 (± 0.99)		2.02 ± (± 0.74)	< 0.001
CAL [mm]	3.15 (± 1.98)		1.53 (± 1.81)	< 0.001

*NS* not significant, *NA* not available

Annotation: American Academy of Periodontology (AAP): Stage I—incipient periodontitis, Stage II—moderate periodontitis, Stage III—periodontitis with potential for additional tooth loss, Stage IV—advanced periodontitis with extensive tooth loss and potential loss of dentition.

**Fig. 3:**
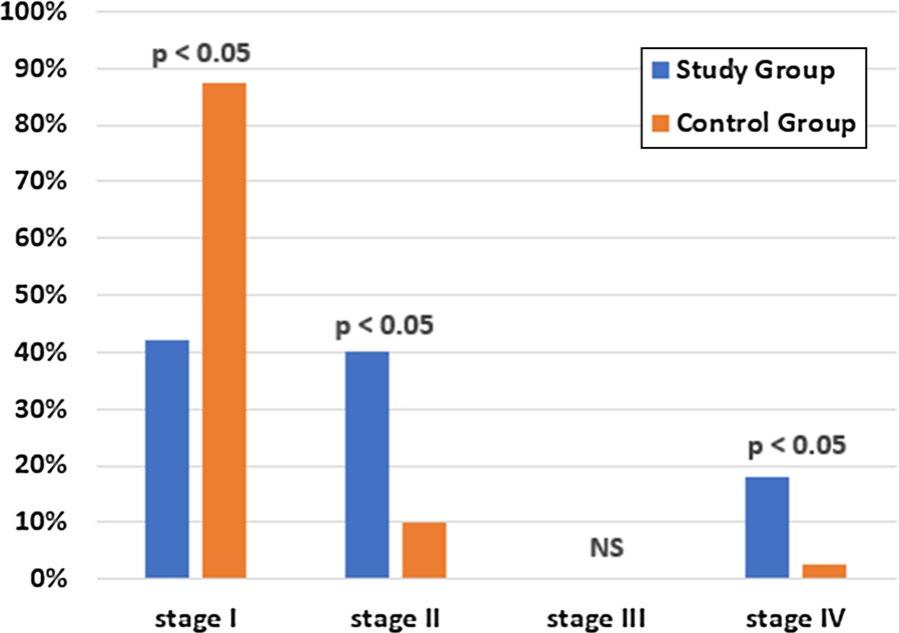
Comparison of the study and control group according to the diagnosis of the AAP

Significant differences were found in the prevalence of stage I periodontitis in favor of control group (87.5% vs. 42%; *p* < 0.05), while advanced periodontits were more prevalent in study group (18% vs. 2.5%; *p* < 0.05) (Fig. 3).

### Periodontological indices and inflammatory biochemical markers

An analysis of the relationship between periodontological indices and inflammatory biomarkers showed that most of the evaluated indices were significantly associated with hsCRP and leukocytes levels. The BoP was statistically significantly associated with the level of fibrinogen in the study group. All indices regarding the pocket depth (PD, NoPD ≥ 4 mm, %PD ≥ 4 mm) correlated significantly with the number of leukocytes. PD and NoPD ≥ 4 mm were also associated significantly with the level of hsCRP. There was also a statistically significant relationship between CAL and hsCRP levels in the study group. However, the number of teeth preserved was associated with ESR (Table 2).

**Table 2:** Spearman's rank correlation for the selected periodontal parameters with the values of inflammatory parameters in the study group

Parameters from dental examination	Inflammatory parameters	The study group n = 71	
		correlations	
		R	*p*
API [%]	hsCRP [mg/l]	R = 0.17	*p* = 0.15
	ESR [mm/h]	R = 0.21	*p* = 0.11
	Leukocytes [G/L]	R = 0.08	*p* = 0.50
	fibrinogen [g/L]	R = 0.11	*p* = 0.42
BoP [%]	hsCRP [mg/l]	R = 0.14	*p* = 0.25
	ESR [mm/h]	R = 0.06	*p* = 0.62
	Leukocytes [G/L]	R = 0.14	*p* = 0.24
	Fibrinogen [g/L]	**R** = **0.36**	***p*** = **0.006**
PD [mm]	hsCRP [mg/l]	**R** = **0.28**	***p*** = **0.01**
	ESR [mm/h]	R = 0.16	*p* = 0.22
	Leukocytes [G/L]	**R = 0.27**	***p*** = **0.02**
	Fibrinogen [g/L]	R = − 0.009	*p* = 0.94
NoPD ≥ 4 mm[n]	hsCRP [mg/l]	**R = 0.24**	***p*** = **0.04**
	ESR [mm/h]	R = 0.07	*p* = 0.59
	Leukocytes [G/L]	**R = 0.28**	***p = 0.02***
	Fibrinogen [g/L]	R =− 0.002	*p* = 0.98
%PD ≥ 4 mm[%]	hsCRP [mg/l]	R = 0.23	*p* = 0.05
	ESR [mm/h]	R = 0.16	*p* = 0.22
	Leukocytes [G/L]	**R = 0.28**	***p = 0.01***
	Fibrinogen [g/L]	R = 0.001	*p* = 0.99
CAL [mm]	hsCRP [mg/l]	**R = 0.27**	***p = 0.02***
	ESR [mm/h]	R = 0.07	*p* = 0.59
	Leukocytes [G/L]	R = 0.10	*p* = 0.42
	Fibrinogen [g/L]	R = 0.04	*p* = 0.74
Number of teeth preserved	hsCRP [mg/l]	R = 0.03	*p* = 0.76
	ESR [mm/h]	**R =− 0.31**	***p = 0.01***
	Leukocytes [G/L]	R =− 0.08	*p* = 0.50
	Fibrinogen [g/L]	R =− 0.20	*p* = 0.13

Bold numbers indicate statistically significant values

### Periodontal indices and markers of heart injury and function

Analysis of the relationship of periodontological indices with biochemical markers of heart injury and function showed few significant correlations. It has been shown that the BoP is correlated closely with the levels of BNP in both the study group. The other analyzed periodontological parameters did not correlate with parameters of myocardial injury and function (Table 3).

**Table 3:** Spearman's rank correlation for the selected periodontal parameters with the values of cardiac injury and function parameters in the study group

Parameters from dental examination	Cardiac injury and function parameters	The study group	
		correlations	
		R	*p*
API [%]	BNP [pg/ml]	R = 0.07	*p* = 0.56
	TnI [ng/ml]	R =− 0.22	*p* = 0.07
	CK-MB [U/l]	R = − 0.126	*p* = 0.31
	Left ventricle ejection fraction [%]	R = 0.23	*p* = 0.08
BoP [%]	BNP [pg/ml]	**R = 0.29**	***p = 0.02***
	TnI [ng/ml]	R = 0.17	*p* = 0.17
	CK-MB [U/l]	R = 0.108	*p* = 0.387
	Left ventricle ejection fraction [%]	R = − 0.15	*p* = 0.26
PD [mm]	BNP [pg/ml]	R = 0.17	*p* = 0.19
	TnI [ng/ml]	R = 0.09	*p* = 0.49
	CK-MB [U/l]	R = 0.007	*p* = 0.952
	Left ventricle ejection fraction [%]	R = − 0.06	*p* = 0.63
NoPD ≥ 4 mm [L]	BNP [pg/ml]	R = 0.12	*p* = 0.35
	TnI [ng/ml]	R = 0.04	*p* = 0.73
	CK-MB [U/l]	R = − 0.001	*p* = 0.998
	Left ventricle ejection fraction [%]	R = − 0.02	*p* = 0.85
PD ≥ 4 mm[%]	BNP [pg/ml]	R = 0.21	*p* = 0.11
	TnI [ng/ml]	R = 0.08	*p* = 0.51
	CK-MB [U/l]	R = 0.057	*p* = 0.647
	Left ventricle ejection fraction [%]	R = − 0.05	*p* = 0.68
CAL [mm]	BNP [pg/ml]	R = 0.16	*p* = 0.21
	TnI [ng/ml]	R = 0.02	*p* = 0.89
	CK-MB [U/l]	R = 0.062	*p* = 0.618
	left ventricle ejection fraction [%]	R = 0.02	*p* = 0.86
number of teeth preserved	BNP [pg/ml]	R = − 0.18	*p* = 0.15
	TnI [ng/ml]	R =− 0.08	*p* = 0.54
	CK-MB [U/l]	R = − 0.112	*p* = 0.368
	Left ventricle ejection fraction [%]	R = 0.01	*p* = 0.90

Bold numbers indicate statistically significant values

### Multifactorial analysis

A multifactorial analysis was performed in order to determine whether the analyzed periodontological indices are independently related to the myocardial infarction occurrence and markers of heart injury and function.

#### Model of prediction of myocardial infarction

In order to determine the model explaining the probability of occurrence of myocardial infarction, logistic regression analysis was performed using the backward elimination method, taking into account the likelihood ratio, where the explained variable was the occurrence of myocardial infarction, and predictors: number of teeth, API, BoP, PD, CAL. The Hosmer Lemeshow test turned out to be irrelevant, which indicates a good fit of the model to the data. Important predictors of myocardial infarction are API and BoP (Table 4). With an increase in API by 1%, the probability of having a heart attack increases by 8% (OR = 1.08), while with a 1% increase in BoP, the probability of having a heart attack decreases by 7% (OR = 0.93).

**Table 4:** Logistic regression for the prediction model of myocardial infarction

Parameter	B	SE	Wald	OR	95% CI	*p*
Number of teeth	− 0.10	0.06	2.84	0.90	0.80–1.02	0.092
API [%]	0.07	0.02	15.98	1.08	1.04–1.12	< 0.001
BoP [%]	− 0.07	0.02	10.83	0.93	0.89–0.97	0.001
PD [mm]	0.82	0.48	2.93	2.26	0.89–5.76	0.087

The model was adjusted for demographic and clinical confounders: age, sex, medications, smoking, and diabetes

B—non-standardized regression coefficient, SE—standard error, Wald—Wald test value, OR—odds ratio, 95%CI— confidence interval, *p*— significance level

**Fig. 4:**
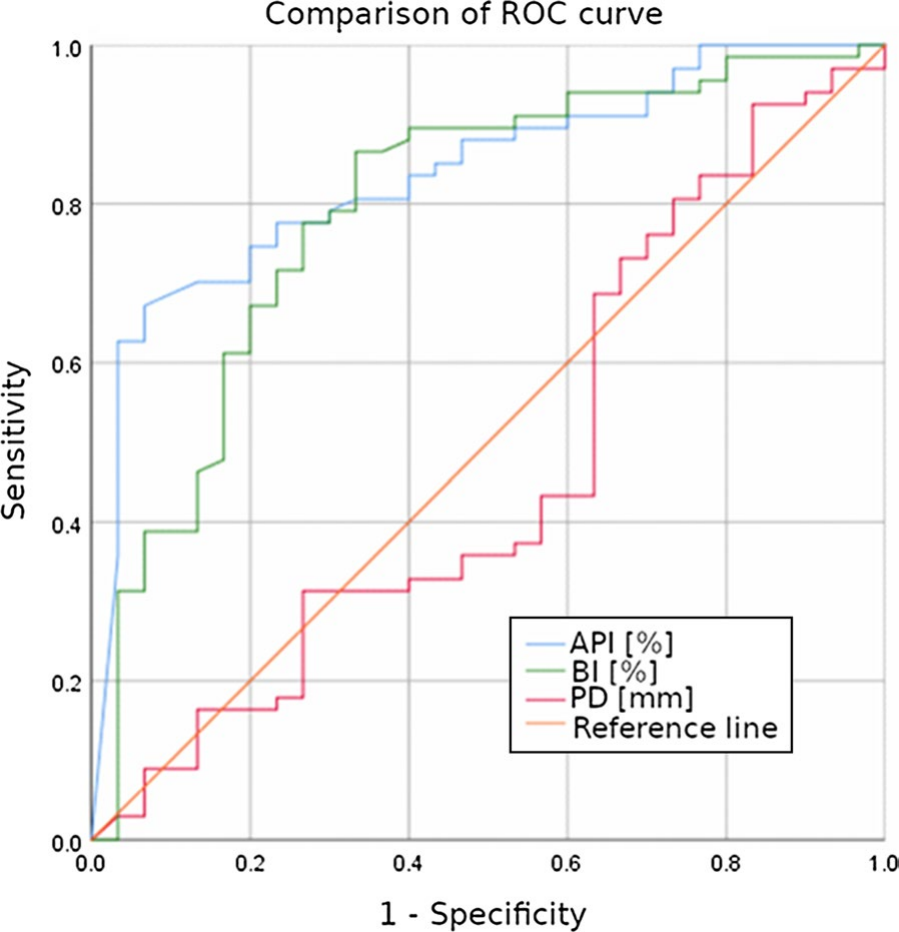
ROC curve for the prediction of the occurrence of a myocardial infarction

The ROC analysis was used to show the performance of the proposed model of prediction of myocardial infarction. The ROC analysis was applied to assess the quality of the classification of the occurrence or the lack of occurrence of a myocardial infarction based on the following three variables: API, BoP and PD. Figure 4 illustrates the ROC curves, while Table 5 summarizes the results of the analysis.

**Table 5:** Summary of ROC curve for the prediction of the occurrence myocardial infarction

Variables	Sensitivity	1 − Specificity	AUC [95% CI]	*p*
API [%]	0.672	< 0.001	0.84 [0.76–0.92]	< 0.001
BoP [%]	0.866	< 0.001	0.80 [0.69–0.91]	< 0.001
PD [mm]	0.910	0.669	0.47 [0.34–0.60]	0.669

AUC—area under curve, 95%CI—confidence interval, *p*—significance level

Based on ROC curves for the group with and without myocardial infarction we concluded that API and BoP have the diagnostic value, while PD proved to be insufficient to present satisfactory prognostic value. In the case of both variables, that is API and BoP, the AUC value indicated very good classification, while for the PD, the classification didn’t differ from random.

The evaluation of the classifier was performed in reference to the Gini coefficient and the Kolmogorov–Smirnov test. The curve was generated on the basis of the variables. For the API the sensibility was 67.2% and the correct prediction of 86.6% cases that is the indication of myocardial infarction patients was possible due to the BoP value.

All metabolic syndrome related markers are of an increased level in the study group. Considering that, the association of periodontal parameters and the acute myocardial infarction may be linked with the metabolic syndrome, therefore an additional analysis adjusted for the metabolic syndrome related markers was performed (Table 6). This analysis confirmed that regardless of the fact that although metabolic syndrome markers (*p* = 0.001 for API and *p* = 0.006 for BoP) indicated a slightly lower statistical significance both the API and BoP, the predictors were important from the perspective of myocardial infarction. With an increase in API by 1%, the probability of the occurrence of a heart attack increases by 7% (OR = 1.07), while with a 1% increase in BoP, the probability of the occurrence of a heart attack decreases by 5% (OR = 0.95).

**Table 6:** Logistic regression for the prediction model of myocardial infarction

Parameter	B	SE	Wald	OR	95% CI	*p*
Number of teeth	− 0.11	0.08	2.77	0.91	0.78–1.03	0.12
API [%]	0.09	0.03	16.22	1.07	1.03–1.11	0.001
BoP [%]	− 0.08	0.04	11.18	0.95	0.9–1.00	0.006
PD [mm]	0.91	0.63	3.01	2.51	0.64–6.01	0.11

The model was adjusted for: age, sex, medications, smoking, diabetes, BMI, triglyceride, HDL-C, blood pressure, and fasting blood glucose

B—non-standardized regression coefficient, SE—standard error, Wald—Wald test value, OR—odds ratio, 95% CI—confidence interval, *p*—significance level

#### Model of troponin level prediction

To check which of the parameters of periodontitis affect TnI levels, a linear regression analysis was carried out by means of backward elimination. In the model, predictors were introduced: number of teeth, API, BoP, PD, NoPD ≥ 4 mm, CAL. The analysis showed that API and BoP are important predictors of TnI levels. With 1% increase in API, TnI levels decrease by 0.3 units, while with 1% increase in BoP, TnI levels increase by 0.26 units (Table 7).

**Table 7:** Linear regression of the TnI level prediction model

Variables	B	SE	t	95% CI	*p*
Constant	43.65	9.05	4.82	25.54 to 61.75	< 0.001
API [%]	− 0.30	0.11	− 2.73	− 0.52 to − 0.08	0.008
BoP [%]	0.26	0.11	2.30	0.03 to 0.49	0.025

The model was adjusted for demographic and clinical confounders: age, sex, medications, smoking, and diabetes

B—non-standardized regression coefficient, SE—standard error, t—t test value, 95%CI—confidence interval, *p*—significance level

#### BNP level prediction model

The next step focused on establishing a model explaining the BNP level. For this purpose, linear regression analysis using backward elimination was used. Model includes the number of teeth, API, BoP, PD, NoPD ≥ 4 mm, CAL as predictors. Linear regression analysis by backward elimination showed that only CAL is a significant predictor (Table 8). As the CAL increases by one unit, the BNP value increases by 31.33 units.

**Table 8:** Linear regression of the BNP level prediction model

Variables	B	SE	t	95% CI	*p*
Constant	37.03	37.27	0.99	− 37.75 to 111.81	0.325
CAL [mm]	31.33	10.20	3.07	10.86 to 51.80	0.003

The model was adjusted for demographic and clinical confounders: age, sex, medications, smoking, and diabetes

B—non-standardized regression coefficient, SE—standard error, t—t test value, 95%CI—confidence interval, *p*—significance level

#### Left ventricle ejection fraction prediction model

For the left ventricle ejection fraction prediction, none of the analyzed models were statistically significant (F < 1.62; *p* > 0.210) and none of the analyzed coefficients were significant left ventricle ejection fraction predictors (*p* > 0.05).

## Discussion

The main assumption of the thesis that periodontitis affects the initiation and progression of atherosclerosis is the fact that the periodontal disease cause subtle systemic inflammation. The present study has revealed elevated levels of inflammation markers in the study group. Leukocytes count was 10.82 (± 3.87) 10^9^/L, ESR 28.69 (± 17.62) mm/h, the levels of hsCRP 8.34 (± 18.72) mg/L, and fibrinogen 4.62 (± 1.09) g/L. In the conducted analysis, a correlation was revealed between the values of periodontal parameters attesting to the presence and the severity of a periodontal disease and the blood level of inflammation parameters. Moreover, there was an inverse correlation between the number of teeth and ESR (R = − 0.31; *p* = 0.01). This may be due to the fact that periodontal inflammation is the main reason for teeth loss.

An increased level of systemic inflammatory response markers, such as leukocytes count, and the levels of hsCRP and fibrinogen, is significant in the pathogenesis of cardiovascular diseases. Determining the levels of these markers may be important in assessing the risk of an acute coronary syndrome. In the experimental models, the influence of CRP on the onset of endothelial dysfunction, increasing expression of adhesion molecules, and on the recruitment of monocytes into the vessel wall was demonstrated. Moreover, CRP contributes to the production of foam cells, formation of reactive oxygen species and to the proliferation and migration of smooth muscle cells [23]. The systematic review and meta-analysis of the study on the correlation between periodontitis and CRP levels conducted by Paraskevas et al. [24] demonstrated elevated CRP levels in blood serum of patients with periodontitis in comparison to healthy people. It was also shown that, among patients with stable coronary artery disease, elevated CRP level increases the risk of myocardial infarction, while in patients with myocardial infarction, it contributes to an increased risk of complications and worse prognosis [25–27]. A multicentre Stability Study showed a link between inflammation markers like hsCRP and interleukin-6 and periodontitis. In this study a large number of patients were collected, however, unlike the present study, the simplest indicator of periodontal disease, which is a tooth loss, was used [28]. In present study, hsCRP level correlated with the parameters attesting to the advancement of the periodontal disease, such as: PD (R = 0.28; *p* = 0.01), NoPD ≥ 4 mm (R = 0.24; *p* = 0.04); moreover, there was a positive correlation with the clinical attachment loss CAL (R = 0.27; *p* = 0.02). Different results were obtained by Górski et al., who did not demonstrate any correlation between CRP levels and periodontal markers [29]. However, Swaroop et al. [30], demonstrated in their study statistically significantly higher levels of inflammation markers, such as hsCRP and fibrinogen, among people with chronic periodontitis than among those with the healthy periodontium (*p* < 0.001); what is more, they proved positive correlations between hsCRP levels and fibrinogen and the measured periodontal parameters (PD, BoP, CAL).

Another extremely important result of the present study is the relationship between periodontal disease and fibrinogen. Namely, a positive correlation between the values of the BoP marker, indicating active inflammation of periodontium, with the level of fibrinogen (R = 0.36; *p* = 0.006). Fibrinogen is a protein synthesized by hepatocytes and fibroblasts in response to inflammation. The level of fibrinogen in the blood correlates with the severity of atherosclerotic lesions, the risk of an acute coronary syndrome, and mortality among patients with myocardial infarction. Fibrinogen participates in the thrombotic process; it is also pro-inflammatory, as it increases the expression of adhesion molecules and stimulates production of inflammatory mediators by endothelial cells [31]. This has an additional significance in the studied group of patients with acute myocardial infarction, where prothrombotic hyperactivity is a key pathogenetic factor. The study by Bokhari et al. [32], in which 317 patients with coronary artery disease and periodontitis were examined, indicated correlation between BoP and the level of fibrinogen. Also Górski et al. [29] documented positive correlation between the concentration of fibrinogen and the values of BoP (*p* = 0.0587), as well as between the number of lost teeth and the level of fibrinogen (*p* = 0.0003). Seringec et al. [33], in turn, demonstrated considerably higher levels of hsCRP, fibrinogen, and globulins among patients with chronic periodontitis, as well as a higher tendency of erythrocytes to aggregate than in people with healthy periodontium.

The present study has demonstrated correlation between the leukocytes count and the parameters indicating the severity of the periodontal disease, such as PD (R = 0.27; *p* = 0.02), NoPD ≥ 4 mm (R = 0.28; *p* = 0.02), as well as %PD ≥ 4 mm (R = 0.28; *p* = 0.01). Numerous epidemiological studies showed positive correlation between leukocytes count and the risk of coronary artery disease. In a prospective NHAHES I epidemiological study, a group of people with the leukocytes count < 6600 cells/mm^3^ was compared with a group with the leukocytes count > 8100 cells/mm^3^; it was found that increased leukocytes count is linked to an increased risk of coronary artery disease among white males (RR = 1.31; 95% CL 1.07–1.61) and white females (RR = 1.31; 1.05–1.63) aged 45–74, taking into account other cardiovascular risk factors [34]. In the meta-analysis of seven most important studies regarding the correlation between the leukocytes count and coronary artery disease, which encompassed 5337 participants with ischemic heart disease, the difference between the leukocytes count below or equal to 2800 cells/mm^3^ was connected to the total RR of 1.4 [35].

Bearing in mind the main purpose of the research, the correlation between periodontical markers and myocardial injury and heart failure indicators was also important observation. Univariate analysis has demonstrated a significant relationship between BoP and the level of BNP in the study group (R = 0.29; *p* = 0.02). Linear regression analysis using backward elimination showed that a significant predictor of BNP is only CAL. As the CAL increases by one unit, the BNP value increases by 31.33 units. For the left ventricle ejection fraction prediction, none of the analyzed models were statistically significant (F < 1.62; *p* > 0.210) and none of the analyzed variables were a significant predictor of left ventricle ejection fraction (*p* > 0.05). BNP is recognized as a prognostic marker in patients with acute coronary syndromes. It is believed that BNP inhibits the growth of cardiomyocytes and fibroblasts, impairs collagen synthesis in relation not only to the myocardium but also to periodontal tissues [36]. Current results seem to confirm the thesis that periodontitis is associated with the biochemical features of heart failure in the course of myocardial infarction. It probably depends on the size of heart injury, as it was asserted by Marfil-Alvarez. This author indicated the correlation between periodontitis and the size of myocardial infarction. This observation is reflected by the higher level of troponin and myoglobin depending on the extent of the myocardial injury. This observation in patients with myocardial infarction is extremely important from a prognostic point of view. Perhaps, an unfavorable prognostic factor in patients with myocardial infarction is not only classic and well-established BNP level but also periodontitis. However, this requires validation studies dedicated to this issue.

An interesting observation is the relationship between the severity of periodontitis and TnI levels. Linear regression analysis showed that significant predictors of the level of TnI are API and BoP. With 1% increase in API, TnI levels decrease by 0.3 units, while with 1% BoP increase, TnI levels increase by 0.26 units. The present research is consistent with the results obtained by Marfil-Alvarez et al., who found a significant correlation between BoP and TnI level (R = 0.21, *p* < 0.025) [37]. Moreover, hierarchical linear regression has showed that the TnI concentration was positively associated with indicators of the extent and severity of chronic periodontitis. Interestingly, the relationship between chronic periodontitis severity and TnI was mediated by the total leukocytes count. On the contrary, current results for patients with acute myocardial infarction are quite different from the data presented by Vedin et al. [28]. Indeed, they found no relationship between periodontal disease, which a simple index was the loss of teeth, and the level of troponin. It should be noticed, however that this study focused on patients with stable coronary heart disease. In earlier studies these authors showed no relationship between tooth loss and myocardial infarction in this population [38].

An equally important and original result of the study is the significant association of periodontitis with risk of myocardial infarction. The logistic regression analysis showed that API and BoP are significant predictors of myocardial infarction. With the increase in API by 1%, the probability of myocardial infarction increases by 8% (OR = 1.08), while with a 1% increase in BoP, the probability of myocardial infarction decrease by 7% (OR = 0.93). The current findings are consistent with the results of the PAROKRANK study of 805 people [39]. A relationship has been demonstrated between moderate to severe periodontitis, objectively confirmed by radiological bone loss, and the first myocardial infarction. Stability Study is a study dedicated to similar topics [28, 38]. In contrast to the our study in PAROKRANK study showed no association with periodontal disease and the onset of the first myocardial infarction. However, while various aspects of cardiovascular risk were assessed in this study, including myocardial infarction, all analyzes were based on a single but very simple indicator of periodontitis which is the number of teeth preserved. Since this was a multicenter observational study, the application of a general parameter that periodontal disease is justified. Remaining in this aspect in a sharp contrast to the present study, it also highlights its originality and methodical credibility. First, it emphasizes the comprehensiveness and diversity of periodontological data collected in the current study. Secondly, it concerns the acute phase of myocardial infarction, which the nature of the disease justifies the difficulties in obtaining so many periodontological data. The fact that the increase in the BoP ratio by 1% is accompanied by a reduction in the risk of heart attack by 7%, also requires comment. Of course, higher BoP indicates a greater severity of periodontitis. It should be remembered, however, that during the dental examination, patients in accordance with acute coronary syndromes treatment standards were already on dual antiplatelet therapy, which undoubtedly increases the risk of bleeding [18, 40]. Moreover, the severity of bleeding may be a clear evidence of the effectiveness of antiplatelet therapy, while it might be a problem in dental treatment in the period after acute coronary syndromes [41]. The explanation for this apparently unexpected relationship can therefore be seen in that—it is a net effect of the severity of periodontitis and the increased bleeding tendency associated with dual antiplatelet therapy. The metabolic syndrome is another important aspect to consider. It is well known that the syndrome constitutes a risk factor for the occurrence of a heart attack and is closely associated with high mortality [42]. The latter may be additionally connected with the frequent occurrence of the coronary atherosclerosis in patients with the pro-inflammatory and pro-thrombotic state characteristic of the metabolic syndrome.[43]. On the other hand, the metabolic syndrome is also associated with periodontitis and tooth loss [44]. The basic exponents for the metabolic syndrome include: visceral obesity, triglyceride, HDL cholesterol, blood pressure, and fasting blood glucose [45]. All markers related to the metabolic syndrome indicted increased values in the present study group. Considering the metabolic syndrome related markers an adjusted analysis was performed. This analysis confirmed that API and BoP are still important indicators of myocardial infarction, although with a slightly lower statistical significance, when compared to the analysis not taking into account the metabolic syndrome markers (for API respectively: *p* = 0.001 and *p* > 0.001; for BoP respectively: *p* = 0.006 and *p* = 0.001). With an increase in API by 1%, the probability of having a heart attack increases by 7% (OR = 1.07), while with a 1% increase in BoP, the probability of having a heart attack decreases by 5% (OR = 0.95).

To sum up, it should be noted that although the metabolic syndrome related disorders undoubtedly affect the relationship between periodontitis and the risk of myocardial infarction, periodontitis still has an independent influence on the occurrence of a heart attack.

On the basis of the conducted analyses, it may be concluded that periodontitis is a condition which may affect the risk of the development of ischaemic heart disease as well as its complications in the form course of myocardial infarction, as it causes a mild systemic inflammatory response. Undoubtedly, social awareness of the possible clinical implication of periodontitis is insufficient. Considering the prevalence of the ischaemic heart disease, high mortality resulting from cardiovascular diseases, and the ubiquity of periodontitis in the Polish society, the periodontal health of the patients with ischemic heart disease should be taken into account and appropriate preventive and curative measures ought to be introduced. Moreover, patients with periodontitis should have their cardiovascular risk assessed. Focusing on any possible correlation between periodontal inflammation and the occurrence of ischaemic heart disease is of utmost importance due to the fact that this may be a modifiable risk factor. The importance of chronic periodontitis should be taken into account in both primary and secondary prevention of cardiovascular disease [46, 47]. What is more, even a single additional tooth brushing episode per day in healthy adult patients can reduce the incidence of atherosclerotic cardiovascular disease events [5]. However, the available literature does not provide sufficient evidence to support or refute the potential benefit of periodontitis treatment in secondary prevention of cardiovascular disease [47, 48]. Undoubtedly, further trials are necessary to conclude whether or not the periodontal disease treatment can help prevent the occurrence or recurrence of cardiovascular disease. According to the recently consensus report published in 2020, patients with periodontitis should be informed about the higher risk for cardiovascular diseases, such as myocardial infarction or stroke, and as such, they should actively manage all their cardiovascular risk factors (smoking, exercise, excessive weight, blood pressure, lipid and glucose management, and sufficient periodontal therapy and periodontal maintenance) [47].

### Strength and limitations

The obvious and permanent limitation of studies with extensive research methodology is a relatively small number of patients. For two reasons, the undoubted indubitable limitation is also used pharmacotherapy for two reasons. Firstly, the use of statins, which is a known anti-inflammatory agent, may modify the severity of the systemic inflammation. However, patients with acute myocardial infarction have not yet used statins in most cases, and blood samples were taken before the drug was started. In turn, antiplatelet drugs may by their nature increase bleeding, including those associated with stomalotological assessment. The more that periodontal examination was performed 24 h after the onset of myocardial infarction and thus anti-platelet activity was fully developed. For ethical reasons, however, this restriction cannot be eliminated because it is not possible to delay the treatment of myocardial infarction for periodontal assessment. It should be emphasized, however, that both in the case of statins and antiplatelet drugs the limitation resulting from their use loses some strength due to the fact that almost all patients had the same treatment. It proves the pharmacological uniformity of the examined group, which increases the reliability of the results. Another limitation is also possible coexistence of other inflammatory processes in patients from the study group. To this end, special care was taken to exclude from the study patients who, at the time of inclusion in the physical examination or in additional examinations, had additional, beyond the periodontium, detectable foci of inflammation.

One of the most important factors allowing the collection of accurate results is probing reproducibility. The factors affecting the repeatability of the assessment may be as follows: measurements taken by different dentists, variable pressure during the examination, the use of different types of periodontal probes. Study looked at intra-examiner and inter-examiner reproducibility for threshold probing depths of greater than 1 mm and found an accuracy rate of 91.3% [49]. Probing depths can be affected by whether a tapered probe or a parallel-sided probe is used, with the parallel probe tending to result in deeper probing depths [50]. However, when both types were compared, 89% of the results showed no difference. In the present study, all dental assessments were performed by one person using one type of probe. It is can be perceived both as a limitation of the study since the same subjective aspects of the assessment were repeated, and also the strength of the study because it determines the uniformity of the assessment in the entire study group. Since any dental evaluation, apart from the measurable numerical values, has a certain amount of subjectivity which cannot be avoided.

The cardiovascular risk factors considered in the study were the commonly recognized risk factors for periodontal diseases. The common risk factors for both diseases include: smoking, diabetes, male gender, age, obesity, dyslipidemia and increased CRP and fibrinogen levels. The multitude of common risk factors makes it difficult to adjust into the multifactorial analysis, which should be considered a study limitation.

In order to avoid possible bias on the part of people conducting individual examinations (dental, echocardiography, coronary angiography), the examinations were performed independently of each other, and the investigators did not have access to the test results obtained by others. Moreover, in order to standardize the dental examination, it was performed by one person. It may also be considered a limitation of the examination since the dental assessed by another independent investigator.

An undoubted advantage of the study is the extensive dental methodology using many periodontological indices. Both the detailed assessment of the periodontal status and the multitude of periodontal indices applied in the study can guarantee an increased reliability of statistical analysis of the obtained results. These studies can increase the interest in the prevention and treatment of periodontal disease in order to improve both the periodontal status and the prevention of cardiovascular diseases in the population.

## Conclusions

- Patients with acute myocardial infarction have worse periodontal status compared to people without coronary heart disease.
- Higher severity of periodontal disease, poorer oral hygiene and increased activity of the periodontitis lead to greater manifestation of systemic inflammation in patients with acute myocardial infarction.
- Periodontitis is a risk factor for myocardial infarction and also affects the degree of post- infarction left ventricular damage, which means that there is an inflammatory link between these two pathogenetically inflammatory diseases.

## Data Availability

The datasets used and/or analysed during the current study are available from the corresponding author on reasonable request.

## Abbreviations

AAP: American Academy of Periodontology
API: Approximal plaque index
BMI: Body mass index
BNP: Brain natriuretic peptide
BoP: Bleeding on probing
CAL: Clinical attachment loss
CK-MB: Creatine kinase myocardial band
CRP: C-reactive protein
ESR: Erythrocyte sedimentation rate
GFR: Glomerular filtration rate
HbA1c: Glycated hemoglobin
HDL-cholesterol: High-density lipoprotein cholesterol
hsCRP: High sensitivity C-reactive protein
LDL-cholesterol: Low-density lipoprotein cholesterol
MI: Myocardial infarction
N/L ratio: Neutrofiles to lymphocytes ratio
NoPD ≥ 4 mm: The number of bleeding periodontal pockets ≥ 4 mm in depth
NSTEMI: Non ST-elevation myocardial infarction
PD: Pocket depth
STEMI: ST elevation myocardial infarction
TG: Triglycerides
TnI: Troponin I
TSH: Thyroid stymulating hormone
%PD ≥ 4 mm: The percentage of bleeding periodontal pockets ≥ 4 mm in depth
